# Supplementary material for: Collaborative intelligence in AI: Evaluating the performance of a council of AIs on the USMLE
Source: PLOS Digit Health. 2025 Oct 9;4(10):e0000787. doi: 10.1371/journal.pdig.0000787 (PMC12510544; doi:10.1371/journal.pdig.0000787)
Supplement: S2 File — (ZIP) [file pdig.0000787.s002.zip › S2 Data Raw data files containing unprocessed question inputs and LLM outputs including transcripts of deliberations/STEP3_Question-90-1_Type-MC-NJ.docx]

**Facilitator** **[Round 1]:** A 28-year-old man comes to the office for an annual health maintenance examination. He says he has been generally healthy. Three months ago he began walking three times weekly for at least 30 minutes, and he has eliminated salt from his diet after a blood pressure measurement at a local pharmacy was 160/96 mm Hg. Medical history is unremarkable and he takes no medications. Family history is remarkable for hyperlipidemia, hypertension, type 2 diabetes mellitus, and coronary artery disease. The patient is 188 cm (6 ft 2 in) tall and weighs 135 kg (298 lb); BMI is 38 kg/m². Vital signs are temperature 36.8°C (98.2°F), pulse 102/min, respirations 18/min, and blood pressure 156/98 mm Hg. The abdomen is protuberant with no masses. The remainder of the physical examination discloses no abnormalities. Results of fasting laboratory studies are shown: Serum Cholesterol Total - 202 mg/dL HDL - 33 mg/dL LDL - 137 mg/dL Triglycerides - 158 mg/dL Glucose - 104 mg/dL Blood Hematocrit - 46% Hemoglobin - 15.6 g/dL WBC - 9800/mm³ Pharmacotherapy should be directed toward which of the following? (A) Aiding with metabolism of glucose (B) Decreasing pulse rate (C) Decreasing serum LDL-cholesterol concentration (D) Lowering blood pressure (E) Suppressing appetite

**AI_C:** RESTATING THE QUESTION: We have a 28-year-old man who has been generally healthy but noted hypertension at a local pharmacy. He made lifestyle changes such as physical activity and eliminating salt from his diet. However, his blood pressure remains high. He has significant family history including dyslipidemia, hypertension, diabetes, and coronary disease. His BMI is classified as severely obese, and he has elevated LDL cholesterol and glucose within the high-normal range. The query is about which pharmacotherapy should be prioritized in this scenario. PLANNING: The assumptions here are that this case is a representation of metabolic syndrome due to his obesity, family history, high blood pressure, and LDL cholesterol, and borderline fasting glucose. This syndrome comprises abdominal obesity, insulin resistance leading to hyperglycemia, lipid disturbances, and hypertension. The other way to see it could be that hypertension is the primary issue, given it’s the parameter that's perturbed most from the normal range, and he already attempted lifestyle modifications without success. After evaluating these, hypertension seems to be the current major clinical issue to address. CLINICAL REASONING: Let's step through the clinical reasoning here. First, if the patient's BMI and family history suggest metabolic syndrome, it means that long-term, this patient is at risk for type 2 diabetes and cardiovascular disease. But his lipid profile and blood glucose, while not optimal, are not outrageously high and still potentially manageable with lifestyle changes which he has started. The most alarming finding is his hypertension, which remains significantly high despite efforts to improve it with lifestyle changes, this might be due to a combination of genetics and the effects of obesity. CONCLUDING: Despite the constellation of borderline disturbances in lipids and glucose, the clinically pressing issue for which pharmacotherapy is most urgent in this case is hypertension. This is due to its severity and persistance, despite lifestyle modifications. RESPONSE: Therefore, the patient would benefit most from pharmacological therapy aimed at (D) Lowering blood pressure.

**AI_D:** RESTATING THE QUESTION: This question highlights the case of a 28-year-old man with multiple family risks for cardiovascular diseases like hyperlipidemia, hypertension, and type 2 diabetes mellitus. He has started to make lifestyle changes due to a high blood pressure reading. Despite this, his current physical examination reveals that his blood pressure remains elevated and his Body Mass Index (BMI) is classifying him as obese. His most recent lab results show his total cholesterol, LDL, triglycerides, and glucose values are within the borderline range. The query is looking for the ideal target of pharmacological intervention. PLANNING: Before proceeding, several assumptions need to be addressed. First, his elevated blood pressure is a cause for health concern, considering the persistent high readings. Second, he already plays an active part in managing his condition through lifestyle modifications. Third, his family history points to genetic predisposition that might require more thorough management than lifestyle changes alone. Fourth, his lab results are within limits but close to the borderlines, which may hint at progressing conditions. Each of these assumptions can influence the direction of the pharmacotherapy intervention. CLINICAL REASONING: Given the patient’s profile and lab results, his elevated blood pressure continues to be a concern. Although he has started making lifestyle modifications, his BMI still classifies him as obese and his blood pressure remains high. These two, in combination with his family history of cardio-metabolic conditions, disproportionately increase his risk of developing cardiovascular diseases. Despite being within limits, his LDL cholesterol is on the higher margin of normal. While they are not primary concerns at this point, any further increase may compound his high blood pressure's effects, potentially accelerating atherosclerotic changes. However, with time, continuous lifestyle modification could improve these borderline parameters. The most urgent concern right now is to control his blood pressure to prevent hypertensive emergencies or complications. CONCLUDING: Given the patient’s history, physical examination, and lab results, the immediate concern should be effective blood pressure management. While his cholesterol, glucose levels, and appetite are areas to address, their immediate intervention is less urgent compared to his persisting high blood pressure. Consistent high blood pressure can lead to serious complications like stroke or renal dysfunction. RESPONSE: The best pharmacotherapy given the case's immediate priorities should be directed towards (D) Lowering blood pressure.

**AI_A:** The question regards a 28-year-old man who now has high blood pressure despite adopting healthier lifestyle habits. His family history is significant for various metabolic diseases, and he is obese. His lab results show slightly high LDL cholesterol but normal glucose levels. We're asked to identify the best pharmacotherapeutic focus out of possibilities including improving glucose metabolism, slowing pulse, reducing LDL cholesterol, reducing blood pressure, or suppressing appetite. First, we need to identify key aspects of the patient's presentation that could guide our selection. This man has an elevated blood pressure reading on two separate occasions, making a diagnosis of hypertension likely. His BMI of 38 kg/m2 falls within the obesity range. Moreover, his family history shows a strong predisposition toward metabolic and cardiovascular diseases. Despite his getting slightly above normal LDL cholesterol level, his fasting glucose is normal, meaning he currently shows no indication of any glucose metabolism disorder. Considering these features, we can make several assumptions. One could be that because of his family history and slightly raised LDL cholesterol level, he may develop hyperlipidemia and a pharmacotherapeutic effort should be targeted toward decreasing his LDL cholesterol concentration. Another assumption could be, given his significant obesity, a pharmacological approach to appetite suppression might be effective in helping him lose weight, which in turn could lower his blood pressure. However, since he has established hypertension, it's also reasonable to think that efforts should be directly aimed at reducing blood pressure. When weighed against the patient's more immediate health problems, the assumption that high LDL cholesterol should be our primary focus is less compelling, mainly because it's just slightly above the acceptable range. As for appetite suppression, while beneficial, it doesn't address his current hypertension, and clinically, blood pressure control is a priority over weight loss in the management of hypertension. Linking back to clinical reasoning, hypertension is a significant risk factor for cardiovascular diseases like stroke, heart attack, and heart failure. Timely treatment is often crucial to prevent these complications. Lifestyle modifications like a low-salt diet and physical exercise have already been initiated by the patient but were unfortunately insufficient, as his blood pressure remains high, reinforcing the necessity of pharmacologic therapy. In summary, though all the given options have a degree of relevance to the patient's overall health status, considering his current health issues, the therapeutic priority should be controlling his blood pressure, which is crucial in reducing his cardiovascular risk. So, the answer is (D) Lowering blood pressure.

**AI_E:** RESTATING THE QUESTION: The question asks us to determine which condition should be primarily targeted by medication in a 28-year-old male patient presenting with a BMI of 38 kg/m², high blood pressure, and laboratory test results indicating slightly increased glucose and LDL-cholesterol. Terms such as BMI (Body Mass Index) and LDL-cholesterol (Low-Density Lipoprotein Cholesterol) are crucial for understanding the health status of the patient. The BMI is often used to ascertain if a person is within a healthy weight range, while LDL-cholesterol is a type of cholesterol that can build up in the arteries and lead to health problems like heart disease. PLANNING: There are several assumptions we need to consider. The patient's increased BMI suggests obesity, which is commonly associated with conditions such as hypertension and hyperlipidemia. Secondly, the presence of slightly elevated glucose can indicate pre-diabetes but may not be alarming as it may also occur in obese patients. The patient's increased LDL concentration, while not extreme, is considered above the ideally recommended level. Finally, the patient's high blood pressure, even after lifestyle modifications, remains a concern. The question is: which should be prioritally targeted? CLINICAL REASONING: Let's follow a stepwise approach to the problem. Obesity can lead to a myriad of health issues, with the immediate concerns being hypertension and the potential development of type 2 diabetes or elevated LDL cholesterol levels, which might lead to coronary artery disease in the future, given this patient's family history. However, the elevation in LDL cholesterol, while above ideal limits, does not appear to be drastic enough to warrant immediate attention. The glucose level is not alarmingly high either, which may not indicate a clear-cut case of impaired glucose metabolism at this stage. The notable problem here is that despite three months of lifestyle modifications, including regular exercise and dietary changes, the patient's blood pressure remains significantly elevated. The persistent hypertension, together with the patient's obesity and family history of hypertension, suggests that controlling blood pressure should be the primary goal of pharmacotherapy at this time. CONCLUDING: Considering all the clinical findings, the priority should be to control his blood pressure as it is persistently high despite dietary modifications and increased exercise. This could prevent possible complications including stroke, heart disease, and kidney disease, to name a few. RESPONSE: Hence, pharmacotherapy should be directed toward (D) Lowering blood pressure.

**AI_B:** RESTATING THE QUESTION: The question presented here is about a 28-year-old male patient who, despite leading a seemingly healthy lifestyle for the past three months, shows certain medical signs such as a high BMI, above normal blood pressure and a protuberant abdomen. His medical history does not present any specific disorder but a family history of hyperlipidemia, hypertension, type 2 diabetes mellitus, and coronary artery disease is notable. We are given his laboratory results which are within normal range for the most part, except for slightly elevated glucose and LDL levels. The challenge is to determine which pharmacotherapeutic action should ideally be the priority considering his condition. PLANNING: Let's identify the key elements in the given information. First, his BMI at 38 kg/m² indicates obesity which is a known risk factor for a number of health issues such as hypertension and diabetes. Secondly, despite some lifestyle modifications, his blood pressure is persistently high around the mark of 156/98 mm Hg. His slightly high serum glucose and LDL levels are also concerning considering his family history of hyperlipidemia and type 2 diabetes. Considering his high BMI, slight glucose elevation, higher LDL levels and hypertension, we need to figure out the most immediate pharmaceutical intervention. CLINICAL REASONING: Given the information, the main area of concern for the patient is his persistent hypertension, which can lead to serious conditions like heart disease and stroke. This cardiovascular risk is further exacerbated by his obesity (high BMI), and family history. The LDL level is slightly elevated, but when considering LDL treatment thresholds, primary consideration is given to overall cardiovascular risk which in this case is primarily being driven by the hypertension. His slightly raised glucose would require monitoring, but is not at a level which requires immediate pharmacotherapy intervention. CONCLUDING: When analyzing the assumption for each response, responses (A) aiding with the metabolism of glucose and (C) decreasing serum LDL-cholesterol concentration don't seem to be immediate priorities given these levels are only slightly elevated. (E) suppressing the appetite could be an approach for long term management of weight but immediate pharmacotherapy could have a direct impact on hypertension. Considering pulse rate, it is slightly elevated likely due to obesity but the direct pharmacotherapy for this might not address the main issue at hand. Hence, it seems that the main situation that needs immediate pharmaceutical intervention would be the patient's hypertension. RESPONSE: Among the given options, the most clinically relevant pharmacotherapy should be directed toward (D) Lowering blood pressure. This direct intervention would address the hypertension issue which currently presents the highest risk for this patient. It would aid in mitigating risks related to his family medical history and could bring further improvements in conjunction with the lifestyle changes that the patient has begun implementing.

**Facilitator** The consensus statement synthesizes the responses of AI_A, AI_B, AI_C, AI_D, and AI_E regarding the pharmacotherapeutic focus for a 28-year-old man with a BMI of 38 kg/m², high blood pressure, and slightly elevated LDL cholesterol and glucose levels. Despite adopting healthier lifestyle habits, the patient's blood pressure remains high. His family history is significant for various metabolic diseases. The key aspects of the patient's presentation that guide the selection of pharmacotherapy include his elevated blood pressure, obesity, family history of metabolic and cardiovascular diseases, and slightly raised LDL cholesterol level. However, his fasting glucose is normal, indicating no current glucose metabolism disorder. While the patient's slightly above normal LDL cholesterol level and significant obesity could suggest a pharmacotherapeutic effort towards decreasing his LDL cholesterol concentration or suppressing his appetite, these assumptions are less compelling when weighed against the patient's more immediate health problems. His LDL cholesterol is just slightly above the acceptable range, and while appetite suppression could help him lose weight and potentially lower his blood pressure, it doesn't directly address his current hypertension. The patient's hypertension is a significant risk factor for cardiovascular diseases like stroke, heart attack, and heart failure. Despite his lifestyle modifications, his blood pressure remains high, reinforcing the necessity of pharmacologic therapy. Therefore, considering the patient's current health issues, the therapeutic priority should be controlling his blood pressure, which is crucial in reducing his cardiovascular risk. Hence, the consensus answer is (D) Lowering blood pressure.
